# Habitats larvaires d*‘Anopheles gambiae* s.l. et mécanismes de résistance à Kribi (Cameroun)

**DOI:** 10.48327/mtsi.v2i4.2022.284

**Published:** 2022-10-25

**Authors:** Patrick NTONGA AKONO, Roméo Serge MBONGUE, Gisèle Aurélie FOKO DADJI, Henri Gabriel TSILA, Léger OFFONO ENAMA, Francis NOPOWO TAKAP, Wolfgang Eyisap EKOKO, Jean Arthur MBIDA MBIDA

**Affiliations:** 1 Faculté des sciences, Laboratoire de biologie et physiologie des organismes animaux, Université de Douala, B.P. 24 157 Douala, Cameroun; 2 École Normale Supérieure de Yaoundé, Laboratoire de Zoologie, Université de Yaoundé I, B.P. 812 Yaoundé, Cameroun; 3 Faculté des sciences, Unité de biologie et écologie appliquée, Université de Dschang, B.P. 067 Dschang, Cameroun; 4 Institut de recherche de Yaoundé (IRY), Organisation de Coordination pour la lutte contre les Endémies en Afrique Centrale (OCEAC), Yaoundé, Cameroun

**Keywords:** *Anopheles gambiae* s.l., Écologie, Gîtes de ponte, Résistance aux insecticides, Mutation *Kdr,* Résistance métabolique, Kribi, Cameroun, Afrique subsaharienne, *Anopheles gambiae* s.l., Ecology, Breeding sites, Resistance to insecticides, *Kdr* mutation, Metabolic resistance, Kribi, Cameroon, Sub-Saharan Africa

## Abstract

**Objectif:**

L'efficacité des moustiquaires imprégnées d'insecticides à longue durée d'action (MILDA) et des aspersions intradomiciliaires (AII) dans le contrôle des vecteurs du paludisme est mise à mal par la résistance des anophèles aux insecticides. Une bonne connaissance des gîtes larvaires ainsi que du profil de résistance des vecteurs pourrait faciliter le développement d'une stratégie appropriée de lutte. La présente recherche s'intéresse à l’écologie larvaire et au niveau de sensibilité d*’Anopheles gambiae* s.l. aux insecticides à Kribi urbain et Kribi rural, localités du sud du Cameroun.

**Méthodologie:**

Les gîtes de moustiques ont été typés et géo-référencés. Pour chaque gîte, les larves ont été prélevées puis mises en élevage et les paramètres physico-chimiques mesurés *in situ.* La sensibilité des vecteurs au dichlorodiphényltrichloroéthane (DDT), à la deltaméthrine et à la perméthrine, après pré-exposition au pipéronyl butoxide (PBO) ou non, a été évaluée sur les anophèles issus de l’élevage de larves. La mutation *Kdr* a été détectée en utilisant la méthode Hot Oligonucleotide Ligation Assay (HOLA).

**Résultats:**

Les gîtes naturels d'An. *gambiae* s.l. étaient constitués d'empreintes de pneu (12%, n = 10), de puits non aménagés (5%, n = 4), de mares d'eau résiduelle (57%, n = 48), d'empreintes de pas et de sabots, de rigoles, de ruisseaux et des berges de la rivière Kienké (15%, n = 13). Les gîtes artificiels étaient constitués de pirogues abandonnées (11%, n = 9). Ces gîtes étaient caractérisés par des valeurs moyennes de température, de conductivité, de salinité et de turbidité plus élevées dans les gîtes enregistrés en zone urbaine par rapport à la zone rurale. Les tests de sensibilité montraient que la mortalité était moins élevée en situation d'absence de pré-exposition au PBO qu'en situation de pré-exposition au PBO dans les deux zones d’étude pour le DDT et la deltaméthrine. La fréquence de l'allèle résistant (R) était élevée pour la mutation *Kdr* West aussi bien à Kribi urbain (0,94) qu’à Kribi rural (0,93).

**Conclusion:**

*An. gambiae* s.l. colonise une gamme variée de gîtes dans les sites d’étude et développe des résistances de types métabolique et génétique vis-à-vis des insecticides recommandés. La recherche de molécules alternatives est une nécessité.

## Abstract (en)

**Background:**

The effectiveness of Long-Lasting Insecticidal Nets (LLINs) and indoor residual spraying (IRS) in controlling malaria vectors is hampered by the resistance of anopheles to insecticides. A good knowledge of the breeding sites and of the resistance profile of the vectors could facilitate the development of an appropriate control strategy. This study looks at the larval ecology and the susceptibility profile of *An. gambiae* s.l. to insecticides in urban and rural areas in Kribi, South Region of Cameroon.

**Methods:**

Mosquito breeding sites were categorized and geo-referenced. For each site, larvae were collected and reared and physicochemical parameters were measured *in situ*. The susceptibility of anopheles to dichlorodiphenyltrichloroethane (DDT), deltamethrin and permethrin, after pre-exposure to piperonyl butoxide (PBO) or not, was evaluated on the reared larvae. The *Kdr* mutation was detected using the Hot Oligonucleotide Ligation Assay (HOLA).

**Results:**

Natural breeding sites of *An. gambiae* s.l. were tyre tracks (12%, n=10), unbuilt wells (5%, n=4), pools of residual water (57%, n=48), foot and hoof prints, gullies, streams and the banks of the Kienké River (15%, n=13). Artificial breeding sites were abandoned dugouts (11%, n=9). Breeding sites in urban areas were characterized by higher mean values of temperature, conductivity, salinity and turbidity compared to the breeding sites in the rural area. The breeding sites of *An. gambiae* s.l. in urban Kribi were found to be sunnier than those in rural Kribi. A total of 4320 adults were used for testing, 1 440 mosquitoes from rural Kribi, 1 440 from urban Kribi and 1 440 specimens from the laboratory Kisumu strain. For DDT and deltamethrin, susceptibility tests showed that mortality was lower in a situation of no pre-exposure to PBO than in a situation of pre-exposure to PBO in the two study areas. The frequency of the resistant allele (R) was high for the *Kdr* West mutation in both urban (0.94) and rural areas in Kribi (0.93).

**Conclusion:**

*An. gambiae* s.l. colonizes a wide range of breeding sites and develops metabolic and mutation resistance to recommended insecticides. The search of alternative molecules for vector control is a necessity.

## Introduction

Bien avant la découverte de son agent pathogène en 1880 par Alphonse Laveran, le paludisme a toujours été une maladie redoutable [21]. Cette parasitose est responsable d'environ 241 millions de cas infectés et 627 000 décès en 2021 [25]. L'Afrique subsaharienne apparaît comme la partie du globe la plus touchée avec 94% des cas et des décès [25]. Ces dernières années, la visibilité de la lutte contre le paludisme et l'appui politique dont celle-ci a bénéficié ont considérablement augmenté. De nouvelles stratégies de lutte ont été mises en œuvre par les programmes nationaux, parmi lesquelles la lutte antivectorielle occupe une place de choix. Cette approche préventive utilise principalement les moustiquaires imprégnées de pyréthrinoïdes et les pulvérisations intradomiciliaires d'insecticides à effets rémanents [15]. Initialement, les techniques d'imprégnation utilisées entraînaient une perte relativement rapide d'efficacité des moustiquaires imprégnées d'insecticide (MII) et nécessitaient un retraitement régulier à l'aide de solutions insecticides [18]. Des avancées récentes ont permis l'essor d'une nouvelle génération de moustiquaires imprégnées à longue durée d'action (MILDA) ou *Long-Lasting Insecticidal Nets* (LLINs) qui ne nécessitent pas de ré-imprégnation. La présence de l'insecticide fixé à l'aide d'une résine à l'intérieur des fibres permet la conservation de leur efficacité pendant 3 à 5 années et/ou après de nombreux lavages [16]. Cet outil est utilisé aujourd'hui par de nombreux pays à travers les programmes nationaux de lutte contre le paludisme et divers organismes de santé [25]. Cependant, des problèmes de résistance des moustiques aux insecticides perdurent et mettent en péril l'efficacité et la pérennité de cet outil.

Les premiers cas de résistance ont été enregistrés au Burkina Faso avec l'apparition de la résistance d’*Anopheles gambiae* s.l. à la dieldrine, puis un an plus tard au dichlorodiphényltrichloroéthane (DDT) [4]. Concernant les pyréthrinoïdes, l'OMS (1987) a signalé la résistance à ces produits chez *An. arabiensis* au Soudan et chez *An. gambiae* au Nigéria [27]. C'est en 1992 que les premières résistances aux pyréthrinoïdes ont été observées au Cameroun chez les populations naturelles d’*An. gambiae* s.l. [2]. De nombreux autres cas sont décrits en Afrique de l'Est [28], en Afrique de l'Ouest [5] et en Afrique du Sud [11].

La résistance des vecteurs aux insecticides affecte à la fois l’économie et la santé publique et vétérinaire à l’échelle mondiale. Elle oblige à augmenter les quantités d'insecticides utilisées (entraînant donc une hausse des coûts). Elle rendrait peu efficace les produits disponibles et les stratégies de lutte contre les vecteurs, entraînant une prévalence accrue des pathogènes et des maladies qu'ils transmettent [23].

L'enjeu de l'heure auquel les acteurs de lutte sont confrontés consiste à gérer la résistance en vue de définir une stratégie de lutte plus efficiente. Cette gestion passe par une bonne connaissance de l’écologie des vecteurs, de leur sensibilité aux insecticides utilisés dans l'imprégnation des moustiquaires et des mécanismes de résistance développés par ces moustiques. Les informations de cette nature sont disponibles dans quelques régions du Cameroun à l'instar de celle du Centre [7], du Littoral [24, 19] et du Nord [8]. Cependant, malgré les conditions écoclimatiques favorables à la prolifération des vecteurs du paludisme, la localité de Kribi dans la région du sud du Cameroun demeure très peu explorée.

La présente étude se propose de déterminer l’écologie, la sensibilité aux insecticides et les mécanismes de résistance des vecteurs du paludisme à Kribi urbain et Kribi rural, en vue de la mise sur pied d'une stratégie de lutte adaptée aux réalités locales.

## Méthodologie

### Sites d’étude

**Figure 1:**
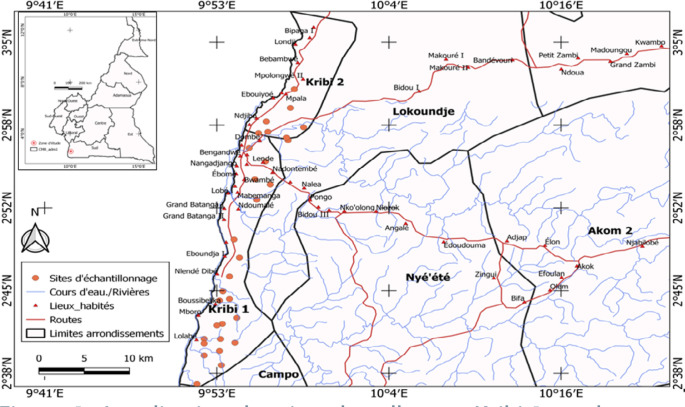
Localisation des sites de collecte à Kribi 1 rural et Kribi 2 urbain Location of sampling sites in Kribi 1 (rural) and Kribi 2 (urban)

La présente étude a été menée à Kribi (03°54’N et 12°31’E), une zone de forêt littorale du sud du Cameroun (Fig. 1). La végétation est riche en césalpiniacées (azobé, padouk, iroko…) et en espèces non ligneuses (lianes, bambou…). Le climat est du type subéquatorial guinéen, réparti en quatre saisons dont une grande saison de pluies (septembre-novembre); une grande saison sèche (décembre-mars); une petite saison de pluies (avril-mai) et une petite saison sèche (juin-août). Les températures moyennes annuelles oscillent entre 26 et 37 °C. L'humidité relative est de 80% et les précipitations moyennes annuelles sont de l'ordre de 2970 mm (Centre météorologique d'Ebolowa, 2018). Deux sites ont été choisis en raison de leurs particularités écologiques: Kribi rural et Kribi urbain.

Kribi rural (02°55’N, 09°55’E) s’étend sur 334 km^2^ pour une population estimée en 2015 à 22 681 habitants [14]. Ce site est en cours d'anthropisation du fait de sa proximité avec le port en eau profonde. Plusieurs édifices majoritairement en matériaux définitifs sont en construction à travers le site, donnant lieu à la destruction du couvert végétal. Les routes, non bitumées pour la plupart et parsemées de nids-depoule, ainsi que la présence d'une grande décharge d'ordures (Hysacam), constituent des facteurs de risque de prolifération des moustiques, surtout en période de pluies. L'on note par ailleurs l'usage de pesticides en agriculture [25]. Les moyens de protection des populations contre les moustiques sont surtout les moustiquaires imprégnées d'insecticide [20], qui ont été gratuitement distribuées aux populations de la localité en 2016 puis en 2019.

Kribi urbain (02°57’N, 09°55’E) s’étend sur une superficie de 125 km^2^ pour une population estimée à environ 40 000 habitants. Ce site est plus anthropisé que le précédent avec une urbanisation mal contrôlée. Le dysfonctionnement des services d'hygiène est à l'origine de nombreux dépotoirs d'ordures qui polluent les potentiels gîtes de moustiques en saison pluvieuse. L'outil de prévention le plus utilisé par les populations contre les piqûres de ces arthropodes est la MILDA. La marque Interceptor imprégnée de l'alpha-cyperméthrine est celle récemment distribuée sur le site.

### Écologie larvaire et identification morphologique des adultes

Les prospections larvaires ont eu lieu aux mois de juin et octobre 2020 ainsi qu'aux mois de janvier et avril 2021. À chaque prospection, les collections d'eau susceptibles de contenir des larves d'anophèles ont été visitées dans une aire d’échantillonnage d'environ 3,5 km^2^ sur chaque site. Chaque gîte répertorié et susceptible d'héberger des larves d'anophèles a été géo-référencé et typé (origine du gîte: permanent, temporaire, sa surface, sa profondeur, transparence de l'eau, distance des habitations, présence de végétation). Les larves ont été collectées de 9 h à 14 h selon la méthode du « dipping » ou « coup de louche » qui consiste à plonger, en plusieurs endroits du gîte larvaire, un récipient de capacité connue. Les paramètres physico-chimiques ont été évalués sur le terrain à l'aide d'un multiparamètre (salinité, conductivité température, pH). Toutes les informations susmentionnées ont été enregistrées sur une fiche conçue à cet effet. Les larves de moustiques ont été élevées séparément en fonction de leur gîte de provenance. Les adultes émergents ont été morphologiquement identifiés [10].

### Tests de sensibilité d'An. *gambiae* s.l. aux insecticides

Après émergence des adultes issus de la collecte larvaire, les tests de résistance ont été réalisés sur des individus âgés de 2 à 5 jours. Au total, trois insecticides (deltaméthrine, perméthrine et DDT) ont été testés conformément au protocole standard [26].

Pour chaque insecticide, ces tests ont nécessité entre 120 et 150 moustiques répartis en 4 répliques (25 moustiques/réplique) et 2 répliques pour le contrôle. Pour ce faire, les moustiques ont été prélevés à l'aide d'un aspirateur à bouche et introduits dans chaque tube d'observation. Après une heure d'observation, chaque réplique de moustiques, à l'exception des témoins a été transférée dans un tube d'exposition aux parois tapissées de papier imprégné d'insecticide. Pendant l'heure d'exposition, le nombre de moustiques assommés *(knocked down)* a été enregistré à chaque fois à 5 minutes d'intervalle. Après l'heure d'exposition, les moustiques ont été transférés dans les tubes d'observation au-dessus desquels ont été déposés des cotons imbibés d'une solution de glucose 10%. Les tests de sensibilité associés aux synergistes ont été réalisés de façon simultanée avec les tests diagnostic mais avec la particularité que chaque réplique de moustiques à tester a d'abord été exposé aux synergistes (pipéronyl butoxide, PBO) pendant une heure. Les moustiques pré-exposés au PBO ont été ensuite exposés à l'insecticide pendant une heure.

Vingt-quatre heures après exposition, la mortalité dans chaque tube a été enregistrée et les moustiques morts ou vivants ainsi que les contrôles ont été conservés séparément dans des tubes Eppendorf contenant du gel de silice en vue des manipulations ultérieures.

À l'issue de ces tests, l'interprétation des résultats se fait comme décrit ci-dessous:

- Une mortalité ≥ 98% signifie que la population est sensible;
- Une mortalité comprise entre 90 et 98% indique une résistance probable;
- Une mortalité < 90% indique une population résistante.

Le test n'est valide qu'en cas de mortalité inférieure à 5% dans le groupe témoin. Néanmoins, si la mortalité du témoin est comprise entre 5% et 20%, la mortalité du test devra être corrigée par la formule d'Abbot (1925) [1].

### Identification moléculaire des membres du complexe *Anopheles gambiae* s.l.

L'identification des espèces du complexe *Anopheles gambiae* s.l. a été faite par la technique de PCR (Polymerase Chain Reaction).

L'extraction d'ADN a été faite au bromure de cétyltriméthylammonium (CTAB) à 2% sur moustique entier.

### Recherche de la présence de la mutation *Kdr*

La recherche de la mutation *Kdr* dans les échantillons d'anophèles a été faite d'après le protocole de Martinez-Torres *et al.* (1998) [17].

### Analyse statistique des données

Toutes les données collectées ont été enregistrées sur un tableur Excel 2016 et transférées dans le logiciel SPSS (Statistical Package for Social Sciences) version 22.0 pour des analyses statistiques.

Le test de Mann-Whitney nous a permis de comparer les valeurs des paramètres physico-chimiques des gîtes de chaque site d’étude.

Le test H de Kruskal Wallis a permis de comparer les taux de mortalité de la souche Kisumu à celui des souches locales.

## Résultats

### Écologie larvaire, typologie et géolocalisation des gîtes

Au total, 84 gîtes larvaires productifs d'anophèles ont été identifiés et prospectés dont 59 à Kribi rural et 25 à Kribi urbain. Les larves des anophèles étaient présentes à la fois dans les gîtes naturels et artificiels. Les gîtes naturels étaient constitués d'empreintes de pneu (12%, n = 10), de puits non aménagés et ensoleillés (5%, n = 4), de mares d'eau résiduelle (57%, n = 48), d'empreintes de pas et de sabots, de rigoles, de ruisseaux et des berges de la rivière Kienké (15%, n = 13). Les gîtes artificiels étaient constitués des pirogues de pêcheurs abandonnées aux débarcadères (11%, n = 9). Les données relatives à la géolocalisation et à la typologie des gîtes larvaires *d'An. gambiae* s.l. sont consignées dans le Tableau I.

**Tableau I:** Typologie et géolocalisation de quelques gîtes naturels et artificiels les plus productifs des larves d'anophèles en zone rurale et en zone urbaine Typology and geolocation of some of the most productive natural and artificial breeding sites of anopheles larvae in rural and urban areas

		Coordonnées géographiques			
	Nom des gîtes	Longitude N	Latitude E	Altitude	Type de gîte
	MP 1	02°55.248‘’	009°55.343”	20 ± 3	Puits
	MP 2	02°55.251”	009°55.317”	20 ± 3	Marécage
	MP 3	02°56.258”	009°54.456”	0	Marre
	MP 4	02°55.154”	009°55.221”	27 ± 3	Empreinte de pneu
**Kribi rural**	TA 1	02°55.157”	009°55.188”	25 ± 3	Empreinte de pneu
	TA 2	02°55.151”	009°55.155”	25 ± 3	Empreinte de pas
	MB 1	02°55.148”	009°55.149”	21 ± 3	Pirogue
	MB 2	02°55.244”	009°55.002”	20 ± 3	Pirogue
	MO 1	02°55.246”	009°54.996”	18 ± 3	Récipient
	MO 2	02°55.103”	009°54.964”	17 ± 3	Empreinte de pneu
**Kribi urbain**	DO 1	02°57.320”	009°55.730‘’	25 ± 2	Mare
	DO 2	02°57.353”	009°55.741”	26 ± 2	Mare
	AF 3	02°57.234”	009°55.756”	24 ± 3	Mare
	AF 4	02°57.180”	009°55.788”	25 ± 2	Empreinte de pneu
	BI 5	02°57.103”	009°55.857”	22 ± 2	Empreinte de pneu
	BI 6	02°57.089”	009°55.857”	21 ± 3	Mare
	ZA 7	02°57.083”	009°55.853”	21 ± 2	Berge de la rivière
	ZA 8	02°57.508”	009°55.582”	20 ± 2	Mare
	NG 9	02°57.591”	009°55.633”	28 ± 3	Mare
	NG 10	02°57.725”	009°55.834”	25 ± 2	Mare

MP=Mpangou, TA= talla, MB=Mboa-manga, MO=Mokolo (Kribi 1er) /DO= Dombè, AF= Afan-mabé, BI=Bissila ZA= quartier zaïre, NG=Ngoye

### Caractérisation physico-chimique des gîtes larvaires

Les gîtes où vivaient les larves d'An. *gambiae* s.l. sont caractérisés par des valeurs moyennes de température, de conductivité, de salinité et de turbidité plus élevées dans les gîtes enregistrés à Kribi urbain par rapport aux gîtes de Kribi rural (Tableau II). Les valeurs moyennes de pH des gîtes à *An. gambiae* s.l. se sont révélées constantes dans les deux sites. Les gîtes où se développaient *An. gambiae* s.l. à Kribi urbain se sont montrés plus ensoleillés que ceux de Kribi rural.

**Tableau II:** Paramètres physicochimiques des gîtes d'anophèles à Kribi urbain et Kribi rural Physicochemical parameters of the anopheles breeding sites in rural and urban Kribi

Paramètres physicochimiques	Site urbain	Site rural	
**Espèce**	*An. gambiae* s.l	*An. gambiae* s.l	p-value
**pH**	8,06 ± 0,31	8,05 ± 0,26	0,464
**Conductivité (µS/cm)**	181,2 ± 27,8	176,4 ± 22	0,631
**Salinité (ppm)**	97,6 ± 14,6	82,8 ± 10,1	0,396
**Température de l'eau (°C)**	31,3 ± 0,6	29,1 ± 0,5	0,015
**Ensoleillement**	(+++)	(++)	-
**Présence de végétation**	(+)	(+++)	-

µS/cm: micro siemens/centimètre; ppm: partie par million; °C: degré Celsius

### Profil de sensibilité des moustiques aux insecticides et détection du gène de mutation *Kdr*

Au total, 4 320 adultes ont été utilisés soit 1 440 moustiques provenant de Kribi rural, 1 440 provenant de Kribi urbain et 1 440 spécimens provenant de la souche Kisumu de laboratoire.

Les faibles taux de mortalité allant de 3,75% à 67,5% enregistrés avec les doses discriminatoires de deltaméthrine (0,05%), perméthrine (3,75%) et DDT (4%) démontrent l'existence d'une résistance des populations *d'An. gambiae* s.l. aux insecticides à Kribi (Tableau III).

**Tableau III:** Mortalités (%) des femelles d'An. *gambiae* s.l. (souches rurale, urbaine, Kisumu) obtenues après 24h d'observation Mortality (%) of female of An. gambiae s.l. (rural, urban and Kisumu strain) obtained after 24 hours of observation

Souche	Insecticides											
	Perm. 75%	PBO + Perm. 75%	P	St	DDT 4%	PBO+ DDT 4%	P	St	Delta.0,05%	PBO + Delta. 0,05%	P	St
**Rurale**	36,25%	68,75%	0,018	R	3,75%	18,75%	0,017	R	67,5%	85%	0,013	R
**Urbaine**	43,75%	77,5%	0,019	R	6,25%	15%	0,036	R	58,75%	90%	0,019	R
**Kisumu**	100%	100%	1,000	S	77,5%	82,5%	0,225	R	100%	100%	1,000	S

DDT = dichlorodiphényltnchloroéthane, Delta: deltaméthrine; PBO = pipéronyl butoxide, Perm: perméthrine; %mort.: moyenne de 4 répliques en raison de 20 moustiques par réplique; P: probabilité; R: Résistante; S: Sensible; St: Statut

### Effet du synergiste pipéronyl butoxide (PBO) sur la mortalité d’*An. gambiae* s.l. exposés aux insecticides

Après une pré-exposition au PBO, le synergiste a permis de restaurer partiellement la mortalité dans la population d'An. *gambiae* s.l. pour la deltaméthrine, la perméthrine et le DDT (Tableau III). Ce résultat montre bien que le mécanisme métabolique est partiellement impliqué dans la résistance aux insecticides à Kribi, mais traduit également l'implication d'autres mécanismes de résistance tels que les mutations de type *Kdr.*

Un total de 350 moustiques ayant survécu aux tests de sensibilité a été utilisé, afin de rechercher la présence du gène *Kdr* qui confère une résistance croisée aux pyréthrinoïdes et DDT. Deux types de mutation *Kdr* ont été trouvés au sein des populations d’*An. gambiae s.l.* Dans les deux zones, la fréquence de l'allèle résistant (R) (0,93-0,94) était élevée pour ce qui est de la mutation *Kdr* West tandis quelle était faible (0,15-0,18) pour ce qui est de la mutation *Kdr* East (Tableau IV).

**Tableau IV:** Fréquences alléliques des mutations Kdr chez les échantillons d'An. *gambiae* s.l de Kribi *Table IV: Allelic frequencies of Kdr mutations in* An. gambiae *s.l. samples of Kribi*

Fréquences alléliques				
Sites	Taqman	N	R	S
**Kribi urbain**	Kdr West	100	0,94	0,06
	Kdr East	100	0,18	0,82
**Kribi rural**	Kdr West	75	0, 93	0,07
	Kdr East	75	0,15	0,85
	**Total**	**350**	**-**	**-**

S: allèle kdr sensible; R: allèle résistant; N: nombre d'individus analysés

## Discussion

La présente étude avait pour but de déterminer l’écologie larvaire ainsi que la sensibilité d’*An. gambiae* s.l. aux insecticides utilisés en santé publique à Kribi (sud du Cameroun).

Il ressort de ces travaux que les larves d’*An. gambiae* s.l. prolifèrent dans une grande variété de gîtes. Ces gîtes étaient temporaires, de nature artificielle ou naturelle. À Kribi rural, les gîtes identifiés étaient tous naturels et largement dominés par les mares d'eau résiduelles ensoleillées et les empreintes de pneu. L'on note l'absence des sociétés industrielles dont les déchets peuvent constituer des gîtes artificiels. Ce résultat est conforme à ceux enregistrés dans plusieurs sites ruraux d'Afrique [13]. Ces gîtes résultent majoritairement de l'engorgement par les eaux de pluies des nids de poule identifiés sur les routes secondaires non bitumées. Lorsqu'ils ne sont pas perturbés parle passage de véhicules, ces gîtes constituent la véritable cause de prolifération des anophèles dans la localité. Les travaux antérieurs menés par de nombreux auteurs ont montré qu’*An. gambiae* est une espèce héliophile se développant dans les forêts dégradées des zones rurales d'Afrique et dans les périphéries des villes, contrairement à *An. coluzzii* qui a développé des capacités adaptatives aux environnements plus ou moins pollués des milieux urbains [12]. À Kribi urbain, en plus des gîtes naturels, l'on note une forte présence des gîtes artificiels. Ces derniers sont le résultat du caractère insalubre que présente cette localité. La plupart des caniveaux sont bouchés et les poubelles jonchent les rues. Cette situation d'insalubrité est à l'origine non seulement de la prolifération des anophèles, mais davantage des *Aedes* et des *Culex* qui affectionnent les eaux croupies [12]. Les gîtes à *An. gambiae* s.l. se caractérisent par une conductivité électrique, une salinité et une température plus élevées à Kribi urbain qu’à Kribi rural. Les valeurs assez élevées de ces paramètres physico-chimiques renseignent sur le niveau de pollution de ces gîtes et partant, sur l'espèce du complexe *gambiae* susceptible de s'y adapter. Plusieurs études ont montré qu’*An*. *coluzzii* Coetzee & Wilkerson 2013 était la seule espèce du complexe *gambiae* capable de se développer dans de telles conditions physico-chimiques [9].

Les tests de sensibilité ont été réalisés à partir des adultes issus de l’élevage en laboratoire et âgés de 2 à 5 jours. Si ce test est conforme au protocole standard de l'OMS [26], il convient de relever que ce protocole montre certaines limites. En effet, le fait de tester la sensibilité aux insecticides uniquement sur des moustiques âgés de 2 à 5 jours n'est pas représentatif d'une vraie population de moustiques. L'impact sur les moustiques âgés n'est potentiellement pas le même. De plus, ce sont les moustiques âgés qui sont les plus intéressants à évaluer car ils ont l’âge d’être infectieux. Ces considérations seront prises en compte dans nos investigations futures. Les tests de sensibilité ont montré une résistance d’*An. gambiae* s.l. aux pyréthrinoïdes et au DDT aussi bien à Kribi rural qu’à Kribi urbain. L'utilisation du synergiste pipéronyl butoxide (PBO) démontre que le mécanisme métabolique est partiellement impliqué dans la résistance aux insecticides à Kribi [5]. En effet, la mortalité était élevée lorsque les moustiques étaient pré-exposés au PBO, ce qui suppose une forte activité des enzymes de détoxication dont le but est d'annihiler l'effet toxique des insecticides. Par ailleurs, la fréquence allélique de la mutation *Kdr* était très élevée dans tous les sites prospectés. Si les données sont disponibles sur les mécanismes de développement de cette résistance [6], les facteurs à l'origine de la sélection de cette résistance ne sont pas clarifiés et méritent des investigations approfondies. Toutefois, certains auteurs ont estimé que l'utilisation massive des insecticides en agriculture et en santé publique est à l'origine des pressions sélectives conduisant à la sélection de la résistance aux insecticides chez les vecteurs du paludisme [3, 8]. En effet, dans les années 1970, l'on a assisté à un usage massif des pyréthrinoïdes dans les plantations afin de combattre les ravageurs de cultures. Bien plus, depuis 4 décennies, les pyréthrinoïdes sont intensément utilisés contre les moustiques nuisibles et vecteurs du paludisme sous forme de sprays et de moustiquaires. Ces protocoles de lutte, qui ont particulièrement eu lieu dans le sud du Cameroun, permettent d'expliquer en partie cette résistance. Aussi la résistance des vecteurs aux insecticides pourrait-elle s'aggraver d'année en année pour se généraliser dans l'ensemble des pays d'Afrique et avoir un impact sur l'efficacité des MII.

## Conclusion

Cette étude montre une gamme variée de gîtes d’*An. gambiae* s.l. dans les sites prospectés. Le gène *Kdr* en plus de la résistance métabolique a été détecté parmi les spécimens résistants aux insecticides. La persistance des gîtes larvaires dans les localités d’étude peut entraîner l'augmentation des souches résistantes, ce qui rendrait difficile les stratégies de lutte basées sur les molécules actuelles. La recherche de nouvelles molécules aux propriétés insecticides avérées s'avère donc nécessaire.

## Remerciements

Les auteurs remercient les autorités administratives et les populations de Kribi pour leur collaboration.

## Contributions Des Auteurs

Patrick NTONGA AKONO et Roméo Serge MBONGUE ont conçu le projet. Roméo Serge MBONGUE, Francis NOPOWO TAKAP, Léger OFFONO ENAMA et Henri Gabriel TSILA ont réalisé le travail de terrain. Roméo Serge MBONGUE, Gisèle Aurélie FOKO DADJI, Wolfgang Eyisap EKOKO et Jean Arthur MBIDA MBIDA ont réalisé les analyses de laboratoire. Roméo Serge MBONGUE, Patrick NTONGA AKONO, Francis NOPOWO TAKAP et Léger OFFONO ENAMA ont effectué l'analyse des données. Jean Arthur MBIDA MBIDA et Patrick NTONGA AKONO ont supervisé l’étude. Roméo Serge MBONGUE, Patrick NTONGA AKONO, Jean Arthur MBIDA MBIDA et Wolfgang Eyisap EKOKO ont rédigé le projet original. Roméo Serge MBONGUE, Patrick NTONGA AKONO, Jean Arthur MBIDA MBIDA, Gisèle Aurélie FOKO DADJI, Henri Gabriel TSILA et Wolfgang Eyisap EKOKO ont révisé et édité le projet final. Tous les auteurs ont lu et approuvé le manuscrit final.

## Liens D'intérêts

Les auteurs déclarent ne pas avoir de liens d'intérêts.
